# Transcranial Direct Current Stimulation (tDCS) for Depression during Pregnancy: Results from an Open-Label Pilot Study

**DOI:** 10.3390/brainsci11070947

**Published:** 2021-07-19

**Authors:** Anna Katharina Kurzeck, Esther Dechantsreiter, Anja Wilkening, Ulrike Kumpf, Tabea Nenov-Matt, Frank Padberg, Ulrich Palm

**Affiliations:** 1 Department of Psychiatry, Hospital of the University of Munich, 80336 Munich, Germany; annakatharina.kurzeck@t-online.de (A.K.K.); esther.dechantsreiter@med.uni-muenchen.de (E.D.); anja.wilkening@med.uni-muenchen.de (A.W.); ulrike.kumpf@med.uni-muenchen.de (U.K.); tabea.nenov-matt@med.uni-muenchen.de (T.N.-M.); frank.padberg@med.uni-muenchen.de (F.P.); 2 Medical Park Chiemseeblick, Hospital for Psychosomatics, Rasthausstr. 25, 83233 Bernau-Felden, Germany

**Keywords:** depressive disorder, pregnancy, non-invasive brain stimulation

## Abstract

Introduction: Depression is the most common morbidity during pregnancy. Available first-line therapy options are limited and depressive disorders in pregnant women are often untreated, leading to negative effects on maternal and fetal health. Objectives: The aim of this open-label pilot study is to extend evidence on the use of transcranial direct current stimulation (tDCS) as a treatment of antenatal depression and to point out options for the use of tDCS in this population. Methods: Six drug-free female patients with major depressive disorder during pregnancy (later than 10th gestational week) were included in this pilot study. Patients were treated with twice-daily tDCS (2 mA, 30 min, anode: F3, cathode: F4) over ten days during inpatient stay (Phase 1) and with once-daily tDCS over 10 days during an optional outpatient stay (Phase 2). Clinical (HAMD-21, BDI) and neuropsychological ratings (Trail Making Test A/B) were performed at baseline, after two and four weeks as well as an obstetric examination. Results: Six right-handed females (23–43 years, 12–33. gestational week) completed Phase 1; four patients additionally joined in Phase 2. tDCS was well tolerated and no adverse effects occurred. Clinical ratings showed an improvement of mean baseline HAMD-21 from 22.50 ± 7.56 to 13.67 ± 3.93 after week 2, and to 8.75 ± 4.99 after week 4. The mean baseline BDI was 26.00 ± 13.90 and declined to 11.17 ± 5.46 after week 2, and to 9.25 ± 3.30 after week 4. Conclusions: Statistically significant changes in HAMD-21 and BDI were observed after Phase 1. One patient achieved remission in terms of HAMD in Phase 1. Although this small-scale study lacks sham control, it shows clinical improvement and absence of adverse events in this critical population.

## 1. Introduction

Up to 10% of pregnant women suffer from depression. This means that depressive disorders are the most common morbidity during pregnancy. Untreated depression in pregnant women is associated with prenatal and postnatal complications for the child, e.g., small for gestational age (SGA), premature delivery, low Apgar scores, higher risk of mental and developmental disorders in childhood [1,2]. There is a strong link to the development of depression for the mother during the postpartum period [3,4]. This could negatively affect mother-child-interaction [5]. Finally, during maternal depression, changes in hormonal and neurotransmitter homeostasis negatively influence the hypothalamus-pituitary-adrenal (HPA) axis in the fetus and set the prerequisites for chronic stress and dysfunctional coping with negative stimuli in later years of life. This leads to hyperactivation of autonomous nervous system functions, reflected by biomarkers such as blood tension, heart rate, and EEG pattern, which can be modulated by relaxation techniques, e.g., relaxing music [6]. Thus, appropriate treatment of maternal depression is necessary to prevent sustained proneness for chronic stress in the child. However, the two standard lines of treatment for depression during pregnancy—psychotherapy and psychopharmacologic treatment [7,8]—are not free from risk either. Psychotherapy as monotherapy for severe depressive episodes is insufficient as it may take up to several weeks or months until the onset of effects, which leads to a prolonged state of untreated depression [8]. At the same time, antidepressant medication as an established first-line treatment of major depression (Selective Serotonin Reuptake Inhibitors most frequently applied) is effective, but is related to the cause of teratogenic effects on the fetus and adverse effects on pregnancy and birth [9,10,11,12]. Consequently, pregnant women often deny pharmacologic treatment. Taken together, untreated depression as well as the limitations of first-line treatments for depressive disorders during pregnancy may set risks for both mother and child.

Non-invasive brain stimulation (NIBS) techniques like transcranial direct current stimulation (tDCS) have proven to be a suitable therapy option for depressive disorders with a favorable safety profile in non-pregnant patients [13]. tDCS uses weak electrical current to modulate neuronal activity in areas which are supposed to be dysfunctional, i.e., decreased neuroplasticity and altered neurocircuitry activity in the left and right dorsolateral prefrontal cortex (DLPFC) [14,15,16]. Therefore, anodal stimulation of the left and cathodal stimulation of the right DLPFC have turned out to be the target areas for tDCS in depressive disorders.

A fundamental advantage of tDCS is the limitation of the current impact on the patient’s, i.e., maternal, brain without systemic influences and the documented absence of serious adverse effects in thousands of tDCS applications [17]. Mild and transient side effects like headache or pruritus are well accepted by patients [18].

According to available data tDCS is considered as a safe, feasible, cost-effective, and portable treatment method in depressed patients [17,18,19] and could potentially become the ideal treatment option of depression during pregnancy once there is a sufficient level of evidence, as shown by only one case report [20] and a first pilot randomized controlled trial (RCT) [21] which showed promising results. This enforces the need of further evidence on tDCS for depression during pregnancy to incorporate tDCS in the treatment guidelines.

However, literature on the application of tDCS for depressive disorders during pregnancy is sparse up to now, as indicated by a systematic review [22].

## 2. Materials and Methods

### 2.1. Study Design

The aim of this open-label pilot study, registered with the German Clinical Trials Register (DRKS00008537), was to gather pilot data on tolerability and efficacy of tDCS to treat major depression in pregnant women. Sham control was omitted for ethical reasons in this particularly vulnerable population and the local ethics committee approved the study protocol. The study design was adopted from an earlier study, which combined antidepressant treatment with an intensified stimulation protocol and a two-stepped inpatient/outpatient setting [23].

This study also consisted of a combined inpatient/outpatient treatment: In the first part (Phase 1, inpatient), patients received tDCS twice a day, i.e., 20 stimulations within two weeks accompanied by standard psychotherapy group sessions twice a week for 90 min each. Phase 1 was followed by an optional second part (Phase 2, outpatient) of two weeks with a single stimulation per day, i.e., 10 stimulations in total during Phase 2. In total, when undergoing both phases, 30 stimulations were applied within four weeks.

### 2.2. Inclusion and Exclusion Criteria

Patients were recruited between 2015 and 2019 at the Outpatient Departments of Psychiatry and Gynecology/Obstetrics of the Hospital of the University of Munich. Pregnant women presenting with clinical symptoms of depression were screened for eligibility and offered participation in the study if they refused first-line pharmacological or psychotherapeutic intervention.

Inclusion criteria: females with a pregnancy of at least 10 weeks’ gestational age; patients between 18 and 45 years old; diagnosis of major depressive disorder, moderate or severe, without psychotic features, as defined in the Diagnostic and Statistical Manual of Mental Disorders (DSM-V) and International Classification of Diseases (ICD-10) criteria.

Exclusion criteria: (1) alcohol or substance use disorder at trial enrolment; (2) acute suicidality; (3) major and unstable medical or neurologic disease; (4) history of traumatic-brain injury or seizure; (4) indication of possible structural abnormalities of brain ganglia or brain stem; and (5) electrical implants in the cranium or neck, except cardiac pacemakers. We also excluded women with (6) a current fetal anomaly or diagnosed obstetrical complication. 

Six females were included after giving written and oral informed consent. All patients declined antidepressant intake prior to enrolment.

### 2.3. tDCS

Constant direct current was applied with a CE-certified Eldith-DC-stimulator (NeuroCareGroup, Munich, Germany): the anode was placed over the left DLPFC (F3, according to the international 10–20 EEG system); the cathode was located over the right DLPFC (F4). Saline-soaked sponge electrodes (7 × 5 = 35 cm^2^) were fixed with rubber bands to the head. Current strength was set to 2 mA and the duration of each stimulation was 30 min plus 15 s fade-in/fade-out.

### 2.4. Rating Instruments

At baseline, the Hamilton Depression Rating Scale-21 (HAMD) as primary outcome and Beck Depression Inventory (BDI) as secondary outcome were evaluated.

Furthermore, Edinburgh Handedness Inventory (EHT), cognitive improvement (Trail Making Test parts A and B, TMT-A/B), general symptom assessment (WHO Quality of Life Bref, WHOQOL), and Clinical Global Impression (CGI) were assessed. The primary endpoint was the number of participants achieving response (≥50% reduction in HAMD) and remission (≥7 in HAMD) at the end of Phase 1 and 2.

Side effects were measured by the Comfort Rating Questionnaire (CRQ) [24]. This self-rating questionnaire assesses side effects (pain, tingling, burning, fatigue, nervousness, disturbed concentration, disturbed visual perception, headache) during and immediately after stimulation (sum scores) and general discomfort on a 10-point Likert scale ranging from “not at all” to “extremely.” Furthermore, the occurrence of light flashes (phosphenes) and sleep disturbances after stimulation was scanned in a dichotomous question.

Clinical ratings were repeated at the end of Phase 1 and Phase 2. Final ratings of Phase 1 corresponded to baseline ratings of Phase 2. A follow-up as part of the regular prenatal care checkups was performed up to the time of birth.

### 2.5. Statistics

For statistical calculation, analysis of variance (ANOVA) was performed with R (R Project for Statistical Computing). Data of patients who started treatment and completed the phases 1 (and 2) including completed questionnaires were used for calculations. Baseline demographic and clinical characteristics were described as mean and standard deviation.

A repeated measures analysis of variance was carried out separately for each rating instrument (HAMD, BDI, CGI, TMT-A/B, and WHOQOL). Missing data were not imputed. Clinical data means were compared using a two-tailed paired *t*-test for dependent samples. The significance level was set at 0.05.

## 3. Results

### 3.1. Clinical and Demographic Characteristics

All six patients completed Phase 1 of the study; four patients completed Phase 2. One patient had to quit the study in Phase 2 after day 18 with 25 stimulations in total due to elevated liver enzymes and fetal intrauterine growth restriction. As further follow-up, a healthy baby (Apgar-score 8/10/10) was delivered without complications after 38 + 5 weeks spontaneously.

Another patient reported sufficient improvement after Phase 1 and renounced Phase 2.

All patients were diagnosed with a (recurrent) depressive disorder. Three showed an episodic course of disease, and three had a continuous form of depression. The number of depressive episodes varied between the first episode (2 patients) and the third episode (2 patients).

An overview of demographic and clinical characteristics is given in Table 1:

### 3.2. Primary and Secondary Outcome Measures

**Figure 1:**
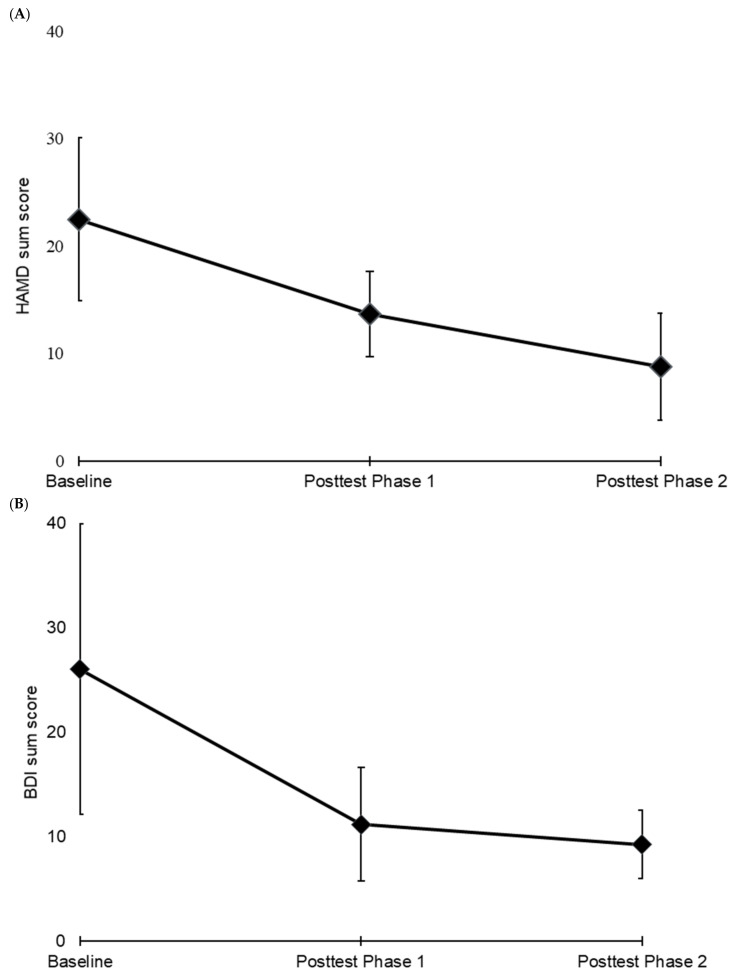
HAMD (**A**) and BDI (**B**) changes.

In Phase 1, significant time effects for all clinical outcomes and a reduction of post-treatment scores in HAMD by 39.26% (mean scores before treatment: 22.5 ± 7.56; after Phase 1: 13.67 ± 3.93, *p* = 0.01, paired *t*-test, 2-tailed) were found (Figure 1A). Two patients (33.33%) achieved response in HAMD rating; none of the patients could achieve remission. Significant time effects were observed for BDI with a reduction by 57.05% (mean scores before treatment: 26.00 ± 13.90; after Phase 1: 11.17 ± 5.46, *p* = 0.04) (Figure 1B). For BDI, two patients (33.33%) achieved response criteria and one (16.67%) achieved remission. CGI improved by 28.57%. WHOQOL dimensions showed no significant time effects, except for the domain “Psychological health” (*p* = 0.04). TMT-A/B results did not change.

In Phase 2, no statistically significant changes could be observed. In terms of HAMD and BDI, one patient achieved remission in each questionnaire, and none achieved response within Phase 2.

Overall, patients undergoing Phases 1 and 2 showed two responses and one remission in terms of HAMD, and one response and one remission in terms of BDI. No significant reductions in HAMD and BDI sum score were noticed. Neuropsychological ratings on the base of TMT-A showed significant time effects (mean scores before treatment: 25.79 ± 4.91; after Phase 2 19.33 ± 3.20, *p* = 0.02). TMT-B showed no significant reduction (*p* = 0.14). WHOQOL showed a significant improvement of the domain “Psychological health”, and CGI indicates a statistically significant change.

Statistical results are summarized in Table 2.

### 3.3. Side Effects and Adverse Events

Over the whole observation period during more than 160 tDCS sessions, only harmless side effects like mild headache during and right after the stimulations, phosphenes, insomnia, and itching sensation beneath the electrodes occurred. The mean CRQ sum score was 19.8 and 14.6 for item 1 and 2, respectively. The mean score of question 3 concerning “general discomfort” of tDCS was 1.5. Two patients indicated sleep disturbances, also mentioning that these probably have to be classified as somatic symptoms of the pregnancy, as they already suffered from sleep problems before enrolment. Three participants perceived phosphenes. Severe adverse effects were not observed, neither in the patients nor in the fetus. Irregularities of fetal and maternal health were not detected during prenatal and neonatal periods in regularly performed check-ups in accordance with the obstetricians, including fetal heart rate measurement. One patient had to quit the study for gestational reasons not related to the stimulation (see Section 3.1).

### 3.4. Follow-Up

Although there was no structured follow-up assessment, we can report the psychiatric outcomes of two patients presented in the further course.

One patient was assessed during a regular outpatient visit after completion of both phases of the study. The final HAMD rating after Phase 2 was 13 and decreased to 8 points within two months. This finding is consistent with existing data about a persistent antidepressant effect of tDCS beyond the acute phase of treatment [25,26].

Another patient, receiving tDCS treatment in this study during her first pregnancy, was treated with transcranial alternating current stimulation (tACS) for recurrent depression in her second pregnancy three years later. In this first case report using tACS [27], the patient had reached remission in the 3-month follow-up (HAMD: 3, BDI: 7).

## 4. Discussion

In this open-label study, we aimed at evaluating the tolerability and efficacy of transcranial tDCS in pregnant women suffering from major depressive disorder. The results showed a significant improvement of HAMD and BDI in six patients undergoing Phase 1 and tDCS was well tolerated without any serious adverse effects. Large effects of improvement were seen in Phase 1 with 20 stimulations in two weeks. HAMD decreased by 39% and BDI by 57% during this first part of the study whereas in the second part, no statistically relevant changes were detected. Interestingly, self-rating by BDI revealed larger improvements than the objective rating by HAMD. This may be explained by the relief of a high personal burden in this patient group and the expectancies raised when receiving a novel intervention. This is also reflected by an improvement of the domain “Psychological Health” of the WHOQOL questionnaire. However, there was no improvement of cognitive functions in the TMT-A/B, pointing out that improvement of mood and cognition after tDCS treatment do not follow a common dosage/effect linearity [28].

In line with the first randomized sham controlled clinical trial published in 2019 [23], our results support the potential benefit of tDCS in the treatment of depression during pregnancy. In the trial by Vigod et al., a direct current of 2 mA was applied to the DLPFC during 15 tDCS sessions. Patients received anodal stimulation over the left DLPFC (F3) and cathodal stimulation over the right DLPFC (F4, according to the 10–20 international system for EEG placement) for 30 min per workday during 3 weeks, i.e., fifteen stimulations in total. Placement of electrodes, current strength, and duration of session are corresponding in both studies. However, the study designs differed. We used a stepped model of twice-daily stimulation during Phase 1 followed by an optional second part (Phase 2) of a single stimulation per day. When completing both phases, participants received 30 sessions in total within 4 weeks. To our knowledge, this is the first study to apply this elevated total charge in pregnant women; albeit repeated twice daily, tDCS was proven to be safe in patients with depression [29]. This intensified treatment regimen with higher total charge applied in a shorter period is in line with the empirical development of tDCS in the last decade, increasing current strength from 1 to 2 mA and prolonging duration from 20 to 30 min per session [30], as there seems to be a dosage-dependent effect on clinical improvement [31].

Our results showed significant HAMD and BDI changes after Phase 1 with 20 tDCS sessions within 2 weeks. Contrarily, Vigod et al. [21] could not observe a statistically significant difference on the antidepressant effect of tDCS using the Montgomery Asberg Depression Rating Scale (MADRS) as the main maternal clinical outcome immediately post-treatment, but significant changes were indicated at the secondary endpoint 4 weeks postpartum. This finding could be due to the open label design in our study, but also due to a higher total charge within the first two weeks [23]. In the Vigod trial, no serious pregnancy or birth complications have been observed in more than 120 tDCS sessions while women treated with tDCS were “satisfied or extremely satisfied with treatment”. These findings concerning the safety profile and tolerability are in line with our results. This kind of satisfaction may be explained by giving care to patients who have constant difficulties in finding adequate treatment for their complaints [32]. Lack of time due to household, children, and work could be another issue in this population [32]. Therefore, self-administered tDCS at home could be helpful to ease access to treatment [33].

### Limitations

Our study has several limitations. First, there is the small sample size. During recruiting, it turned out to be difficult to find and convince patients to participate. On the one hand, depressed pregnant women frequently do not actively search for help as they probably cannot reflect symptoms of depression or cannot confess the disorder in view of the public expectancy of a lucky pregnancy [34]; on the other hand, there might have been reservations against the technique.

Second, the open label design and the lack of sham control hampers quality of the gathered data. It is likely that unspecific effects of care giving, the procedure of tDCS application, the inpatient setting, and the social inclusion by group psychotherapy could have driven the results, at least partially.

Third, there is an insufficient follow-up concerning the maternal and child’s health after birth, and there is no follow-up concerning post-partum depression and mother-child-attachment.

## 5. Conclusions

In summary, the aim of this open-label pilot study was to investigate the efficacy and safety of tDCS as treatment for depression during pregnancy. Here, we applied an intensified treatment regimen with elevated total charge and could show that treatment was safe and led to clinical improvements. Our data is in line with the findings of the first randomized clinical trial and emphasizes the potential of this intervention. tDCS could develop as the first line treatment in antenatal depression due to its easy use, lack of side effects, and its potential for home treatment. Hence, there is need for further clinical trials to collect solid and sound data, as evidence still is very sparse, and study designs are heterogeneous.

## Figures and Tables

**Table 1:** Demographic and clinical characteristics.

	Phase 1 (*n* = 6)	Phase 2 (*n* =4)
Female patients	6	4
Tobacco use	1	0
Handedness (R/L)	6/0	4/0
Years of education (y)	12.8 ± 3.3	13.8 ± 3.4
Mean age (y)	32.5 ± 6.8	30.0 ± 5.6
Age range (y)	23–43	23–35
Age of onset (y)	26.3 ± 4.7	26.0 ± 6.1
Mean gestational week at enrolment	22.8 ± 7.9	18.3 ± 4.3
Range of gestational week at enrolment	12–33	14–21
Course of depression (episodic/continuous)	3/3	2/2
Mean duration of illness (y)	6.2 ± 6.1	4.1 ± 4.8
Number of episodes	2.0 ± 1.0	2.0 ± 1.2
Duration of episodes (months)/range	7.5 ± 10.5/1–26	9.12 ± 11.4/3–26
Total mean duration of hospitalization (months)	1.8 ± 1.9	1.1 ± 1.4
Current mean duration of hospitalization (months)	0.9 ± 2.0	1.1 ± 1.4

**Table 2:** Clinical results in Phase 1, Phase 2, and Phase 1 + 2.

		HAMD	BDI	TMT-A/B		CGI Item 1	WHOQOL					
				TMT-A	TMT-B		Overall Quality of Life	General Health	Physical Health	Psychological Health	Social Relationships	Environmental Quality of Life
**PHASE 1**	Mean Baseline Phase 1 (t = pre-tDCS. *n* = 6)	22.50 ± 7.56	26.00 ± 13.90	28.47 ± 5.98	77.10 ± 36.22	4.66 ± 0.82	3.33 ± 1.21	3.17 ± 0.98	45.24 ± 20.91	37.50 ± 19.72	52.78 ± 27.22	58.34 ± 16.96
	Mean score (t = post 2 weeks tDCS. *n* = 6)	13.67 ± 3.93 (*p* = 0.009) **	11.17 ± 5.46 (*p* = 0.038) *	34.27 ± 23.67 (*p* = 0.564)	76.73 ± 40.06 (*p* = 0.980)	3.33 ± 0.52 (*p* = 0.001) **	4.17 ± 0.41 (*p* = 0.141)	4.33 ± 0.52 (*p* = 0.058)	61.90 ± 20.16 (*p* = 0.105)	58.33 ± 16.24 (*p* = 0.040) *	59.72 ± 20.01 (*p* = 0.419)	63.03 ± 18.37 (*p* = 0.369)
	Change Phase 1 (%)	39.26%	−57.05%	20.39%	−0.48%	−28.57%	25.00%	36.84%	36.84%	55.56%	13.16%	8.04%
	Response/Remission	2 Responses/0 Remission	2 Responses/1 Remission									
**PHASE 2**	Mean Baseline Phase 2 (t = post 2 weeks tDCS. *n* = 4)	13.50 ± 1.29	13.75 ± 2.50	34.33 ± 30.56	52.29 ± 15.74	3.25 ± 0.50	4.25 ± 0.50	4.50 ± 0.58	57.14 ± 21.23	57.29 ± 14.18	64.58 ± 12.50	61.72 ± 17.93
	Mean score (t = post 4 weeks tDCS. *n* = 4)	8.75 ± 4.99 (*p* = 0.113)	9.25 ± 3.30 (*p* = 0.174)	19.33 ± 3.20 (*p* = 0.382)	49.91 ± 16.54 (*p* = 0.379)	3.00 ± 0.82 (*p* = 0.638)	3.75 ± 0.50 (*p* = 0.182)	4.00 ± 0.82 (*p* = 0.182)	71.43 ± 12.37 (*p* = 0.278)	68.16 ± 8.27 (*p* = 0.320)	66.67 ± 26.35 (*p* = 0.809)	67.97 ± 23.44 (*p* = 0.116)
	Change Phase 2 (%)	−35.19%	−32.73%	−43.69%	−4.54%	−7.69%	11.76%	81.86%	25.00%	18.96%	3.23%	10.12%
	Response/Remission	0 Response/1 Remission	0 Response/1 Remission									
**PHASE 1 + 2**	Mean Baseline Phase 1 + 2 (t = pre-tDCS. *n* = 4)	20.75 ± 4.93	26.00 ± 10.30	25.79 ± 4.91	62.90 ± 21.02	4.75 ± 0.96	3.75 ± 0.50	3.25 ± 0.96	42.86 ± 23.87	36.46 ± 12.44	54.17 ± 22.05	54.69 ±17.95
	Mean score (t = post 4 weeks tDCS. *n* = 4)	8.75 ± 4.99 (*p* = 0.058)	9.25 ± 3.30 (*p* = 0.061)	19.33 ± 3.20 (*p* = 0.016) *	49.91 ± 16.54 (*p* = 0.139)	3.00 ± 0.82 (*p* = 0.035) *	3.75 ± 0.50 (*p* = 1.000)	4.00 ± 0.82 (*p* = 0.391)	71.43 ± 12.37 (*p* = 0.165)	68.16 ± 8.27 (*p* = 0.043) *	66.67 ± 26.35 (*p* = 0.495)	67.97 ± 23.44 (*p* = 0.224)
	Change Phase 1 + 2 (%)	−57.83%	−64.42%	−25.04%	−20.65%	−36.84%	0.00%	23.08%	66.66%	86.94%	23.08%	24.29%
	Response/Remission	2 Responses/1 Remission	1 Response/1 Remission									

Note: * *p* < 0.05 ** *p* < 0.01.

## Data Availability

The data presented in this study are available on request from the corresponding author. The data are not publicly available due to privacy reasons.
